# The Reparation of Cranial Defects by Means of Cartilaginous Grafts

**Published:** 1917-11

**Authors:** 


Technique of the Operation :

**The Reparation of Cranial Defects by Means of Cartilaginous Grafts.** By H. L. Warren Woodroffe, Lyon, France. Surgeon to the Ulster Volunteer Hospital. Abstracted from the *British Journal of Surgery*, July, 1917.

Because in military surgery the gap left in the skull after trephining is large and no benefit from it to the patient is derived, a reparative operation is often desirable.

The signs indicating the need of the operation are : visible pulsation and impulse on coughing which result from defective equilibrium of the brain. Symptoms are : headache, vertigo, sudden blurring of vision, especially when induced by quick movements ; also sleeplessness and distress from noise, pain, and giddiness upon slight pressure.

An operation securing a permanent and physiological protection is preferable to any form of head covering, which cannot prevent traumatism of the brain from the edges of the gap, and which becomes wearisome.

Although Jacksonian epilepsy may be caused by other conditions than unstable intracranial equilibrium, an operation is desirable to find and remove the cause. In many cases no cause but the cranial breach has been found and hence a reparative operation is generally desirable. Even the cosmetic effect makes it worth while for frontal gaps.

Besides the usual contra-indications, any bronchitis makes the operation undesirable because of the extreme pain caused by coughing in the lower costal regions from which the cartilage grafts are removed. In no case should the mending be undertaken until the last scab has dropped off from the wound or while there is any marked bulging or any sign of intracranial tension. No gap compatible with life is too large for repair.

Of the possible forms of operation W. prefers cartilaginous grafts because they are safe, simple, autoplastic, and performed with a minimal shock. Besides the grafts are autogenous, involve a minimum of unpleasantness and risk, and permit rapid recovery. The operation may be performed almost immediately after the last crusts have separated. It requires no special plant. Cartilage resists infection. A case is reported where the cartilage survived and solidified after mild suppuration. Cosmetic results are more easily obtained than with other methods.

*Technique of the Operation* :

The author prefers a crucial incision with the cross over the middle of the hole in the skcull, except in wounds involving the temporal muscle.

The plane of dissection is the sub-aponeurotic layer.

Care must be exercised not to injure the dura. The edges of the bone are carefully cleared with a rugine, so that the dura will be separated from the margin of the bone. The fibrous scar replacing the injured part of the dura is best left alone.

Grafts of the cartilage are shaved from the 6th, 7th, or 8th costal cartilage, or the whole tip of a floating cartilage may be taken. Each graft is cut and dropped into warm saline solution.

To hold the grafts in place, a net-work of catgut may be attached to the margin of the pericranium. The hole in the skull may then be filled by sliding the grafts beneath this catgut trellis. The grafts should everywhere overlap. They do not ossify, but form a cartilaginous plate filling the gap of the bony skull.

The indication for the operation is the necessity of protecting the brain from injury due to the continual contact against the edge of the breach, resulting in headache, dizziness, and other symptoms due to insufficient insulation of the brain. "Viewed from this stand-point, the results are very satisfactory".

A review of the experience of Gosset, Villandre, and the author, shows favorable results in the majority of the operations. Marie reports less satisfactory results.

